# Chronic Neuroinflammation in Alzheimer's Disease: New Perspectives on Animal Models and Promising Candidate Drugs

**DOI:** 10.1155/2014/309129

**Published:** 2014-06-16

**Authors:** Christopher Millington, Sandra Sonego, Niloo Karunaweera, Alejandra Rangel, Janice R. Aldrich-Wright, Iain L. Campbell, Erika Gyengesi, Gerald Münch

**Affiliations:** ^1^Department of Pharmacology, School of Medicine, University of Western Sydney, Locked Bag 1797, Penrith South DC, NSW 2751, Australia; ^2^Nanoscale Organisation and Dynamics Group, School of Science and Health, University of Western Sydney, Locked Bag 1797, Penrith South DC, NSW 2751, Australia; ^3^School of Molecular Bioscience and the Bosch Institute, University of Sydney, Camperdown, NSW 2006, Australia; ^4^Centre for Complementary Medicine Research, University of Western Sydney, Locked Bag 1797, Penrith South DC, NSW 2751, Australia; ^5^Molecular Medicine Research Group, University of Western Sydney, Locked Bag 1797, Penrith South DC, NSW 2751, Australia

## Abstract

Chronic neuroinflammation is now considered one of the major factors in the pathogenesis of Alzheimer's disease (AD). However, the most widely used transgenic AD models (overexpressing mutated forms of amyloid precursor protein, presenilin, and/or tau) do not demonstrate the degree of inflammation, neurodegeneration (particularly of the cholinergic system), and cognitive decline that is comparable with the human disease. Hence a more suitable animal model is needed to more closely mimic the resulting cognitive decline and memory loss in humans in order to investigate the effects of neuroinflammation on neurodegeneration. One of these models is the glial fibrillary acidic protein-interleukin 6 (GFAP-IL6) mouse, in which chronic neuroinflammation triggered constitutive expression of the cytokine interleukin-6 (IL-6) in astrocytes. These transgenic mice show substantial and progressive neurodegeneration as well as a decline in motor skills and cognitive function, starting from 6 months of age. This animal model could serve as an excellent tool for drug discovery and validation* in vivo*. In this review, we have also selected three potential anti-inflammatory drugs, curcumin, apigenin, and tenilsetam, as candidate drugs, which could be tested in this model.

## 1. Alzheimer's Disease

### 1.1. Alzheimer's Disease: A Disease without a Neuroprotective Treatment

Dementia is one of the major causes of disability and dependency among older people worldwide. In 2012, 35.6 million people have dementia worldwide and there are 7.7 million new cases reported every year [1]. It has been predicted that the number of people affected by dementia will double every 20 years to 81.1 million by 2040 [1]. Alzheimer's disease (AD) is the most common form of dementia, accounting for up to 70% of all sporadic, late-onset cases of dementia. In an aging population, the number of dementia sufferers increases every year and this poses significant health and social problems for society in the very near future. Despite decades of research dedicated to the development of new pharmaceutical compounds designed to slow down disease progression, no drugs with real disease-modifying properties are available at this point in time. There is a growing consensus in the scientific community that disease-modifying treatments which start before the onset of clinical dementia are needed [2].

### 1.2. Neuropsychology and Pathophysiology of AD

The first behavioural symptoms of AD include significant memory problems in immediate recall and short-term or long-term memory. Additionally there are significant deficits in at least one of four areas: expressing or comprehending language; identifying familiar objects through the senses; poor coordination, gait, or muscle function; and the executive functions of planning, ordering, and making judgments. These symptoms appear gradually and become steadily impaired over time [3].

The main pathological hallmarks of AD are two types of protein deposits, amyloid plaques and neurofibrillary tangles and the degeneration of cholinergic neurons in the basal forebrain and the associated loss of cholinergic neurotransmission in the cerebral cortex and other areas. [4]. Amyloid-beta (A*β*) peptide is the main constituent of senile plaques and creates one of the key pathological features of AD. Although the initial cause of sporadic AD is still debated, the “amyloid cascade hypothesis” states that the aberrant production, aggregation, and deposition of A*β* is a causative process in the pathogenesis of AD [5]. Some researchers have found evidence that A*β* fibrils form pores in neurons, leading to calcium influx and the neuronal death associated with AD [6]. Apart from the direct role in cell death, A*β*-mediated glutamate receptor modifications can lead to synaptic dysfunction, resulting in excitotoxic neurodegeneration during the progression of AD [7]. However, the long list of negative clinical trials targeting amyloid production or deposition compels us yet again to reexamine the amyloid cascade hypothesis as only a marginally significant pathogenic mediator of disease and to perhaps revert back to traditional science principle where repeated negative data leads one to consider other ideas [8–10].

Tau is one of the microtubule-associated proteins (MAPs) that stabilize neuronal microtubules for their role in the development of cell processes, establishment of cell polarity, and intracellular transport. Neurofibrillary protein aggregates are one of the major hallmarks of AD. These aggregates, also known as neurofibrillary tangles (NFT), are formed as a result of abnormal hyperphosphorylation of tau protein, aggregation into “paired helical filaments” (PHFs), cross-linked by reactive carbonyl compounds [11, 12], and loss of ability to maintain the microtubule tracks [13]. Despite a lack of understanding of the intermediate steps involved, NFT formation appears to be caused by aberrant signalling that leads to an imbalance of kinases/phosphatases, resulting in hyperphosphorylation of tau and its detachment from microtubules. Microtubule breakdown follows, an aggregation of tau into PHFs, which in turn bundle into NFTs of “neuropil threads” that then leads to the disintegration of intracellular transport and neuronal degeneration [14].

### 1.3. Current Treatment Strategies for AD

The two major pharmacological treatment options against AD currently available are acetylcholinesterase inhibitors and memantine, a glutamate receptor associated channel blocker. Among those, cholinesterase inhibitors are the most common medications used for the treatment of AD; however these treatments are symptomatic and only temporarily effective [15]. Although antiamyloid drugs were initially hoped to slow down disease progression, all respective clinical trials have failed to yield positive results. As there are no disease-modifying therapies available for the treatment of AD at the moment, alternative targets such as low-grade neuroinflammation are attracting more and more interest in the search of a disease-modifying treatment for AD [16–18].

### 1.4. The Cholinergic Deficit in AD

Cholinergic neurons are one of the most prominent features of the mammalian basal forebrain that can be described as a collection of aggregated and nonaggregated, large, hyperchromic neurons; many of them contain choline acetyl transferase (the key enzyme in the synthesis of acetylcholine) and project to the cerebral cortex and hippocampus [19]. Cholinergic neurons are widely distributed in the basal forebrain, including the medial septum, the nucleus of the horizontal, vertical, and lateral limbs of the diagonal bands of Broca, and the ventral pallidum, in the basal part of substantia innominata, and in the extension of amygdala. Cholinergic input from the basal forebrain to the cortex and the hippocampus has a key role in mechanisms of cognitive functions, including arousal, attention, sensory processing, and memory [20, 21]. The number and size of the basal forebrain cholinergic neurons as well as cortical acetylcholine axon density have been found to decrease with normal aging and in AD [22]. In AD, in contrast to normal ageing, the progression of brain atrophy caused by neuronal and synaptic loss is rapidly accelerated. Massive cholinergic cell death in the nucleus basalis was originally suggested to be one of the major indicators of AD [23], and the resulting cholinergic deficits in the cortical and hippocampal regions have been correlated with the severity of dementia [24]. Patients with AD have a significant decrease of acetylcholine in the cortex and show pathological changes in cholinergic basal forebrain neurons [25]. Recent evidence suggests that atrophy of the cholinergic basal forebrain in AD can be distinguished from normal age-related degeneration even at predementia stages of the disease [26]. These findings considered collectively suggest that cholinergic neuronal cell death in the basal forebrain together with the decline of the cholinergic synapse numbers in the hippocampus and the neocortex is characteristic pathological manifestation of AD. Therefore, animal models of AD need to be able to demonstrate these features to be suitable for disease modelling and drug discovery.

### 1.5. Chronic Neuroinflammation Occurs in Early Stages of AD

AD is associated with an inflammatory response as shown by an increased presence of activated microglia and astrocytes, activated complement proteins, cytokines, and reactive oxygen, nitrogen, and carbonyl species [27–31]. These histochemical studies have been confirmed by imaging studies using positron emission tomography and the peripheral benzodiazepine ligand PK11195 as a marker for activated microglia indicates that activation of microglia occurs already in mild and early forms of AD and precedes cerebral atrophy [32, 33]. Genetic studies on AD further confirm the importance of inflammation. For example, genome-wide association studies have identified three genes that are associated with an increased risk of developing AD, CLU (clusterin), CR1 (complement receptor 1), and TREM2 (triggering receptor expressed on myeloid cells 2) [34]. Furthermore, pharmacological evidence also points to the importance of neuroinflammation for AD pathogenesis. Long-term use of nonsteroidal anti-inflammatory drugs (NSAIDs) has been shown to delay the onset of AD, but randomized trials show no benefit from NSAIDs in AD patients. However, asymptomatic individuals treated with the NSAID, naproxen, experienced reduced AD incidence, after only 2 to 3 years [35]. In summary, these data support the significance of neuroinflammation for AD and gave rise to the hypothesis of the “cycle of self-perpetuating inflammatory neurotoxicity” [36]. (a) Multiple inflammatory triggers can lead to microglial activation. These triggers can be peripheral (such as systemic infections or peripheral chronic inflammation) or central (e.g., caused by beta-amyloid peptide in senile plaques). (b) Activated microglia release neurotoxic factors such as cytotoxic cytokines like TNF-*α* and reactive oxygen/nitrogen species and thus cause damage to neighbouring neurons; direct neurotoxic insults (such as energy depletion and hypoxia) might further weaken neurons and make them more susceptible to microglial attack. (c) These damaged or dying neurons release microglia activators, for example, damage associated molecular patterns (alarmins), resulting in a self-perpetuating cycle of neurotoxicity. In summary, neuropathological, epidemiological, and genetic findings show clear evidence for the involvement of neuroinflammation in the early stages of sporadic AD.

## 2. Shortcoming in the Current Amyloid-Based Mouse Models of AD

Transgenic (Tg) mice that overexpress mutant familial Alzheimer's disease (AD) amyloid precursor protein (APP) genes have contributed to an understanding of dementia pathology and support the amyloid cascade hypothesis. Although many sophisticated mice APP models exist, none comprises all features of AD cellular and behavioural pathology. The greater resilience of transgenic mice to substantial A*β* burdens suggests the A*β* levels and forms that are deleterious to human neurons are not as noxious in these animal models [37]. For example, the APP23 mouse model does not demonstrate all features of the human disease, such as cholinergic axon terminal deficits and extensive cholinergic cell loss in relevant areas such as in the cerebral cortex and CA hippocampal area 1 [38, 39]. Furthermore, these mice do not demonstrate the same variety of proinflammatory markers as human AD patients and generally develop a much weaker neuroinflammatory phenotype [40]. For example, when the Tg2576 mice (containing the Swedish double mutation of human APP) were examined for the expression pattern of various cytokines, only a few IL-6-immunoreactive astrocytes were observed, and iNOS immunoreactivity was completely absent [41]. Moreover, in one of our studies investigating the effect of vitamin D depletion and supplementation with vitamin D enriched mushrooms, there was no clear difference between wild-type and amyloid AD transgenic mice (APPswe/PS1dE9) in terms of learning and memory, despite significant deposition of amyloid plaques observed in the AD mice [42].

In summary, amyloid overexpressing transgenic mice were initially thought to provide a useful model to investigate the mechanisms by which cytokines contribute to the progression of AD, including cognitive decline. However, there is decreasing confidence that this is the best animal model for this purpose. Therefore, a novel model of chronic neuroinflammation with resulting neurodegeneration would be helpful; the GFAP-IL-6 mouse model could be ideal for this purpose.

## 3. The GFAP-IL-6 Transgenic Mouse Model as Novel Model of Chronic Neuroinflammation Relevant for AD

The GFAP IL-6 mouse line was initially generated to investigate cytokine signalling in the CNS. In this model, the murine IL-6 gene (and lacZ) is expressed in astroglia under the transcriptional control of the murine glial fibrillary acidic protein (GFAP) promoter, resulting in brain-specific forced expression of IL-6 [43]. In the wild-type (WT) mouse brain, IL-6 levels are undetectable, while in the GFAP-IL6 transgenic, elevated levels of IL-6 are observed in the cerebellum, the striatum, the hippocampus, the hypothalamus, the neocortex, and the pons, resulting in accelerated age-related structural changes seen within 3–6 months compared to normal aged mice [44]. It has also been shown that the level of transgene-encoded IL-6 expression in the CNS of these transgenic mice is similar to that found in EAE and thus falls within a pathophysiological range [45]. Astrocyte production of IL-6 results in a localised inflammatory state within the CNS with the activation of many acute-phase response genes including *α*1-antichymotrypsin, complement C3, and metallothionein [45]. Cellular changes include the activation of astrocytes and microglia, proliferative angiopathy with blood-brain barrier (BBB) breakdown [46], reduced hippocampal neurogenesis [44], and neurodegeneration which is accompanied by age-related deficits in learning and memory [47]. In many respects, the GFAP-IL6 mouse replicates the structural and functional neuropathology of human neurodegenerative diseases including Alzheimer's disease and HIV-associated dementia [46]. Thus, this transgenic mouse not only highlights the capacity for endogenously produced IL-6 to induce inflammation and neurodegeneration in the CNS but also provides a powerful tool in which to explore the basic mechanisms that underpin IL-6 actions and associated neurodegeneration in the brain.

While it is arguable, which of the major proinflammatory cytokines might be the best to create a suitable animal model providing both severe neurodegeneration and being of relevance for AD, we believe that IL-6 is a particular good candidate. As it serves as one of the inflammatory triggers for the following reasons; IL-6 can be consistently detected in the brains of AD patients but not in the brains of nondemented elderly people [48], and increased IL-6 levels in serum have been shown to differentiate dementia from normal ageing [49]. Additionally, connections between genetic variants of IL-6 and the volume of the hippocampus were analyzed using voxel-based morphometry indicating that the IL-6 allele has a significant role in the development of brain atrophy [50]. Based on preliminary data and previous findings, this IL-6 mouse model provides an exceptional opportunity to investigate the detrimental effects of chronic neuroinflammation on the structure and function of the mammalian brain, paying special attention to its effects on the cholinergic system.

## 4. The IL-6 Overexpressing Mouse as a Drug Validation Model

Animal models like the GFAP-IL-6 mouse can be used to perform preclinical proof-of-concept studies and assess the efficacy, mechanism of action, and safety profile of anti-inflammatory and neuroprotective compounds. In animals treated with these compounds, cognitive, functional, and behavioural tests can be performed and complemented with tissue-based assays, to demonstrate* in vivo* the molecular basis, drug efficacy, and mechanism of action. Based on previous research, we have selected three candidate compounds, curcumin, apigenin, and tenilsetam, to evaluate if anti-inflammatory treatment can slow down the progression of neurodegeneration and the decline in cognitive function in the GFAP IL-6 mouse model, which will be discussed in this review.

## 5. Potential Anti-Inflammatory Drug Candidates for the Treatment of AD

### 5.1. Curcumin

Curcumin is a component of the Indian curry spice turmeric (*Curcuma longa Linn*). Curcumin has been shown to have various antioxidant and anti-inflammatory properties. For example, curcumin was shown to decrease the level of inflammatory mediators such as tumour necrosis factor- (TNF-) *α* and inhibited the production of interleukins (IL) 1, 2, 6, 8, and 12, monocyte chemo attractant protein (MCP), and migration inhibitory protein [51–55]. Curcumin inhibits inflammatory cytokines through a number of mechanisms, with* in vitro* studies indicating that curcumin inhibits the activation of certain transcription factors such as activating protein-1 (AP-1) and nuclear factor kappa B (NF-*κ*B) in stimulated monocytes and macrophages, thereby blocking cytokine gene expression [56, 57]. Furthermore, the pharmacokinetic properties of curcumin are favourable in achieving clinical efficacy. Curcumin crosses the blood-brain barrier and curcumin preparations with enhanced bioavailability (delivered orally) can achieve therapeutic concentrations in the brain [58]. For example, brain levels of the curcuminoids reached concentrations of up to 3 *μ*M for curcumin and up to 6 *μ*M for tetrahydrocurcumin (TC) [59]. These concentrations are indicative of clinical efficacy, since they are in the same range as the tissue concentrations for inhibition of mRNA production of inducible nitric oxide synthase (iNOS)* in vivo*, where the EC_50_ values were determined at 1.2 and 0.701 *μ*M for curcumin and TC, respectively [59].

Lim et al. have tested curcumin for its ability to inhibit the combined inflammatory and oxidative damage in Tg2576 transgenic mice. In this study, Tg2576 mice aged 10 months were fed a curcumin diet (160 ppm) for 6 months. These results indicated that the curcumin diet significantly lowered the levels of oxidised proteins, interleukin- (IL-) 1*β*, and soluble and insoluble A*β*, in addition to plaque burden [58]. Following on from this work, Yang et al. evaluated the effect of feeding a curcumin diet (500 ppm) to 17-month-old Tg2576 mice for 6 months [60]. When fed to the aged Tg2576 mice with advanced amyloid accumulation, curcumin resulted in reduced soluble amyloid levels and plaque burden [60]. Moreover, the effect of curcumin in the brain was demonstrated by its capability to modulate cholinergic neurotransmission and, consequently, improve cognition deficits and memory impairment in aged rats [61].

So far, large clinical trials with curcumin in AD patients have been lacking. However, one small study conducted in Japan reported some interesting results [62]. In this study, Hishikawa et al. described three patients with AD whose behavioural symptoms improved remarkably as a result of turmeric (the spice containing curcumin as major ingredient) treatment [62]. After 12 weeks of the treatment, the total score of the Neuropsychiatric Inventory-brief questionnaire decreased significantly in both acuity of symptoms and burden on caregivers. In one case, the minimental state examination (MMSE) score was up by five points, from 12/30 to 17/30. In the other two cases, no significant change was seen in the MMSE; however, they came to recognize their family within 1 year of treatment. In all cases that had been taking turmeric for more than 1 year reexacerbation of behavioural and psychological symptoms of dementia (BPSD) was not seen. Though it is a small sample size, the three AD cases treated with turmeric suggest a significant improvement of the cognitive and behavioural symptoms, suggesting a probable benefit in the use of turmeric in individuals with AD for BPSD [62].

The reported* in vitro* and* in vivo* studies indicate that curcumin is a suitable compound to target pathways involved in neuroinflammation. However, preparations with enhanced bioavailability need to be developed in order to realize therapeutic concentrations in the human CNS [63].

### 5.2. Apigenin

Apigenin is a dietary flavonoid found in a wide variety of plants, fruits, and vegetables. This polyphenolic compound is particularly abundant in the ligulate flowers of the chamomile plant and in other sources such as celery, parsley, and grapefruit [64]. Previous investigations of the biological activity of apigenin have demonstrated potent antimicrobial, anti-inflammatory, antioxidant, and antitumorigenic properties [65–67]. Evidence from several reports suggests a broad and potent anti-inflammatory activity of apigenin [68, 69]. For example, apigenin has been shown to have inhibitory effects on* in vitro* releasing of several proinflammatory mediators in lipopolysaccharide (LPS) and upregulation of proinflammatory markers in murine and human cell lines. Apigenin strongly decreased levels of IL-6 in LPS activated mouse macrophages [70] and suppressed CD40, TNF-*α*, and IL-6 production via inhibition of interferon gamma (IFN-*γ*) induced phosphorylation of STAT1 in murine microglia [71]. Evidence of its anti-inflammatory properties is also demonstrated in studies that show dose-dependent suppression of the inflammatory mediators nitric oxide (NO) and prostaglandin E_2_, through inhibition of iNOS and cyclooxygenase-2 (COX-2) expression in both microglial and macrophage mouse cells [67, 72]. In human cell cultures, apigenin has been demonstrated to attenuate the release of inflammatory cytokines by inactivation of NF-*κ*B, mediated by suppression of LPS-induced phosphorylation of the p65 subunit [73]. Other effects reported for apigenin include decreasing expression of adhesion molecules [74] and its well-known defensive properties against oxidative stress, such as free radical scavenging and increasing intracellular glutathione concentrations [75]. Apigenin is reported to exert many of its effects through interactions with the signaling molecules in the 3 major MAPK pathways (ERK, JNK, and p38) in both murine and human cell culture models [76–78].

There are very few studies on apigenin in models of neuroinflammation and/or cognitive decline including AD animal models. One recent study by Zhao et al. tested the neuroprotective effects of apigenin in the APP/PS1 double transgenic AD mouse [79]. Four-month-old mice were treated orally with apigenin (40 mg/kg) for 3 months. Results indicated that apigenin-treated mice displayed improvements in memory and learning deficits and a reduction of fibrillar amyloid deposits with lowered insoluble A*β* concentrations, mediated by a decrease in *β*-CTF and BACE1. Additionally, the apigenin-treated mice showed restoration of the cortical ERK/CREB/BDNF pathway involved in learning and memory typically affected in AD pathology. Enhanced activities of superoxide dismutase and glutathione peroxidase were also observed and increased superoxide anion scavenging [79, 80]. Similarly, in another study A*β*-_25–35_-induced amnesia mouse models were treated with apigenin (20 mg/kg), resulting in improvements in spatial learning and memory, in addition to neurovascular protective effects [79]. Other* in vivo* studies with non-AD-related animal models report significant reductions in LPS-induced IL-6 and TNF-*α* production in apigenin pretreated mice [70].

Based on the published literature, only one study in humans had been conducted with apigenin with respect to inflammation or cognitive performance. Forty-two patients with AD (12), PD (17), and MS (13) were included in the study; a formulation containing apigenin was administered to them every 12 hours and they were given appointments every three months for a clinical evaluation and a review of general lab analyses. Subjects were submitted to follow-up evaluations between 3 and 24 months (mean: 8.85 ± 5.99 months). Clinical stabilisation was achieved in all the patients (100%) with MS and the scores on the Expanded Disability Status Scale improved in four patients. Clinical stabilisation was achieved in 17 patients (100%) with PD and improvements in the score on the Unified Parkinson's Disease Rating Scale are in 15 of them. Of the AD patients, 12 attained clinical stabilisation (100%) with an improvement in minimental test in nine cases [81]. In view of the role of oxidative stress and neuroinflammatory processes involved in the pathophysiology of AD, there is evidence to suggest that apigenin is a suitable and promising natural compound to further investigate.

### 5.3. Tenilsetam

**Figure 1:**
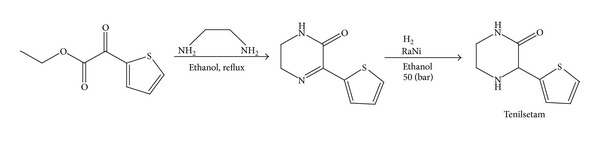
Synthetic route for tenilsetam.

The nootropic drug (±)-3-(2-thienyl)-2-piperazinone (CAS 997, Tenilsetam, Figure 1) has antidementia, antiamnesic, and antihypoxidotic properties.* In vivo*, Tenilsetam has been assayed in human plasma and urine, where recovery in both media was achieved with endogenous background material separated accurately via a HPLC method [82]. Several pharmacokinetic studies were completed and plasma concentrations were observed over 72 h period. With an initial 150 mg dose, the mean plasma concentration reached a maximum value of 3.39 ± 0.64 *μ*g/mL and the mean urine cumulative recovery was 47.3 ± 3.4%. Tenilsetam was absorbed reasonably fast, showing peak plasma concentrations at 2 hours, which were determined to be dose-dependent. The half-life was determined to be between 18 and 22 hours [83].

In human studies, encephalotropic and psychotropic properties were studied in ten aged subjects using quantitative EEG and psychometric analysis. EEG spectral analysis displayed significant CNS activity caused by tenilsetam, where alpha brain activity increased and delta activity decreased. Time-efficacy calculations showed two pharmacodynamic peaks occurring in the 4th and between the 8th and 24th hour. The lag between pharmacokinetic and pharmacodynamic changes is consistent with the hypotheses that tenilsetam needs to penetrate the blood-brain barrier [83].

Several mechanisms of action for tenilsetam have been hypothesized. For example, tenilsetam has been shown to be a carbonyl scavenger (e.g., of methylglyoxal) or inhibitor of advanced glycation end-product (AGE) formation [84, 85]. It inhibits AGE-derived amino acid and protein crosslinking* in vitro* by covalently scavenging toxic reactive carbonyl compounds such as methylglyoxal [86–88]. In addition, mounting evidence suggests that the interaction of AGEs with their receptor RAGE perpetuates inflammation and participates actively in various vascular and inflammatory diseases [11, 27, 89, 90]. There are various RAGE ligands including AGEs, S100 proteins, and amphoterins [91, 92]. It is proposed that tenilsetam could exert an indirect anti-inflammatory action by minimizing proinflammatory AGE formation [84, 89].

*In vitro* experiments with human neuroblastoma SH-SY5Y cells, incubated with methylglyoxal which was preincubated with tenilsetam for 2 or 24 hours, tenilsetam completely nullified the toxicity of methylglyoxal at both time points [87]. A few studies have investigated the effect of tenilsetam in humans in regard to memory and cognition. For example, Wesnes et al. [93] examined the influence of tenilsetam on 18 male undergraduates with the hypothesis that tenilsetam would improve cognitive performance in normal conditions and after scopolamine was administered. In an additional study, healthy volunteers (*n* = 15) received randomized tenilsetam doses (150, 300, and 900 mg) to study proof of antihypoxidotic activity. Blood gas, EEG, and psychometric results were obtained under hypoxic (9.8% O_2_) and normoxic (21% O_2_) conditions. Spectral EEG analysis indicated deterioration in vigilance under the hypoxic conditions, which was attenuated by tenilsetam [94].

Tenilsetam has been used successfully as a treatment in a pilot trial for AD patients. Over a 3-month period at 150 mg/day doses, AD patients showed significant improvements in the favorability judgment task (FJT) and reaction time [95]. The influence of tenilsetam on the P300 response test was also observed with the same dose causing a significant reduction in latency after 4 weeks of treatment [96]. The supporting data surrounding tenilsetam suggest that it would make a suitable compound to test in the GFAP IL-6 mouse to learn more about the drug's mode of action, about its anti-inflammatory and cognitive enhancing properties, and if it attenuates sustained neuroinflammation and prevents cognitive decline.

## 6. Conclusion

Dementia, in particular Alzheimer's disease (AD), poses a substantial challenge to health, aged care, and social policy. Chronic neuroinflammation, demonstrated by the activation of microglia and astrocytes as well as the release of reactive oxygen species and cytokines, has attracted considerable interest in AD over the past decade, not only for its potential role in contributing to neuronal degeneration, but also as a target site for developing potent therapeutics in the future. To investigate the connection between neuroinflammation and AD and to test potential anti-inflammatory drugs, a more suitable “neuroinflammatory” animal model is needed, showing the level of neuroinflammation comparable to the human conditions. Using such animal models, including the GFAP-IL6 mouse line, will allow researchers to investigate the effects of chronic low-grade neuroinflammation on brain structure and function, as well as the effects of anti-inflammatory agents in prevention and recovery.

In this review, we introduced the GFAP-IL6 transgenic mouse, in which the proinflammatory cytokine interleukin-6 (IL-6) is produced by astrocytes under the control of the GFAP promoter, highlighting this as a suitable animal model to study the role of chronic neuroinflammation in neurodegeneration. Previous studies show that these transgenic mice display progressive neurodegeneration in the hippocampal and cerebellar regions and a decline of both cognitive function and motor skills from 6 months of age. It has not been evaluated if the cholinergic system, the neurotransmitter system most affected in AD, is impaired in this model, and it is important to understand if IL-6 induced inflammation may cause cholinergic system degeneration and contribute to cognitive impairment.

Previous studies suggested that curcumin, apigenin, and tenilsetam are promising compounds due to their pharmacokinetic properties and previous success in animal models of familiar AD or in patients. In addition to these three drugs, we anticipate that this animal model can be used to validate a variety of cytokine suppressive anti-inflammatory drugs (CSAIDs) with potential for the prevention and/or treatment of AD from both synthetic and natural sources in the future. The GFAP-IL6 mouse line as animal model of chronic neuroinflammation might be even useful for the investigation and therapeutic intervention relevant for other neuroinflammation related diseases, such as multiple sclerosis, lupus, bipolar disorder, Rasmussen's encephalitis, and traumatic brain injury, which are all associated with microglial activation and neurodegeneration. Furthermore, we believe that this animal model can serve as a valuable* in vivo* bioassay for screening of anti-inflammatory drugs, including the class of CSAIDs, and a successful treatment can be monitored by amelioration of inflammation, neurodegeneration, and cognitive decline.
